# Factors associated with problematic internet use among University of Gondar undergraduate students, Northwest Ethiopia: Structural equation modeling

**DOI:** 10.1371/journal.pone.0302033

**Published:** 2024-06-18

**Authors:** Werkneh Melkie Tilahun, Asefa Adimasu Tadesse, Haileab Fekadu Wolde, Zenebe Abebe Gebreegziabher, Wondwosen Abey Abebaw, Mulat Belay Simegn, Lamrot Yohannes Abay, Tigabu Kidie Tesfie

**Affiliations:** 1 Department of Public Health, College of Medicine and Health Science, Debre Markos University, Debre Markos, Ethiopia; 2 Department of Epidemiology and Biostatistics, College of Medicine and Health Sciences, Institute of Public Health, University of Gondar, Gondar, Ethiopia; 3 Department of Epidemiology and Biostatistics, School of Public Health, Debre Birhan University, Debre Birhan, Ethiopia; 4 Department of Epidemiology and Biostatistics, School of Public Health, College of Medicine and Health Sciences, Woldia University, Woldia, Ethiopia; 5 Department of Environmental and Occupational Health and Safety, College of Medicine and Health Sciences, Institute of Public Health, University of Gondar, Gondar, Ethiopia; National Cheng Kung University College of Medicine, TAIWAN

## Abstract

**Background:**

For young adults and adolescents, excessive internet use has become a serious public health concern due to its negative impact on their health. It has been associated with detrimental effects on both physical and mental health. Negative academic outcomes were observed in the students, including missing classes, lower grades, and academic dismissal. Therefore, the purpose of the current study was to identify factors associated with PIU among undergraduate students at the University of Gondar.

**Method:**

A cross-sectional study was conducted at the University of Gondar among 1514 undergraduate students from June 1–20, 2022. The study participants were selected using a stratified simple random selection procedure. Using structural equation modeling, the degree of relationship was ascertained. A p-value of less than 0.05 and an adjusted regression coefficient with a 95% confidence interval (CI) were used to interpret the data.

**Results:**

In our study, being from non-health departments [β = 0.11, 95% CI: 0.037, 0.181], current alcohol use [β = 0.12, 95% CI: 0.061, 0.187], depressive symptoms [β = 0.23, 95% CI: 0.175, 0.291], insomnia symptoms [β = 0.12, 95% CI: 0.060, 0.196], and ADHD symptoms [β = 0.11, 95% CI: 0.049, 0.166] had a significant positive effect on PIU, while having a history of head injury had a significant negative effect [β = -0.12, 95% CI: -0.226, -0.021] on PIU.

**Conclusion and recommendation:**

Factors such as current alcohol use, non-health department type, depressive symptoms, insomnia, and ADHD symptoms were positively associated with PIU. However, a history of head injuries was negatively associated with PIU. Therefore, strategies aimed at the early identification of PIU may lead to an improvement in the psychosocial health of university students.

## Introduction

Even if the use of the internet has clear and tremendous benefits for the users, it has increased dramatically and shown cases of excessive use, which often has negative health consequences [1]. And currently, it has reached the magnitude of a significant public health concern [1]. The term "problematic internet use (PIU) refers to any use of the internet that causes a person to experience difficulties in their social, academic, professional, or psychological spheres [2]. This problem can occur at any age, in any social, educational, or financial background [3]. This problem also becomes an evident public health problem among university students in Ethiopia [4, 5].

Present-day young adults’ lives revolve around the internet, and excessive use of it has given rise to a new and developing health concern for their health [6, 7]. And it has been linked to detrimental effects on mental and physical health [6, 7]. Moreover, it has also been associated with poor academic outcomes among students, including lower academic grades, missed assignments, and academic disqualification [8]. In general, students with PIU reported poor health behaviors and can lead an unhealthy lifestyle [8–11]. Studies revealed that PIU significantly increased social anxiety [12], eating disorders [9, 11], problematic thoughts [13], suicidal ideation and attempt, social isolation, subjective distress [9, 10, 14, 15], and less health-protective behaviors [9, 11].

Moreover, factors such as age [10, 16–18], sex [5, 9, 19–21], poor sleep quality [22], less frequent exercise [23], year of study [18], marital status [10], residence [24], alcohol consumption [4, 21, 24], perceived social support [24, 25], psychological distress [4, 13, 17, 24], smoking [21], ADHD symptoms [26], depressive symptoms [5, 9, 13, 17, 23, 25, 27], chat chewing, and caffeinated drinks [4, 5] were indicated as significant factors associated with PIU.

University students should pay close attention to problematic internet use, as it is a significant public health issue [15]. Especially in underdeveloped countries, it deserves more exploitation [25]. Studies have shown that additional studies are necessary to fully comprehend the connection between internet use and academic characteristics, as well as physical and mental health [23]. College students’ abilities to evaluate and carry out PIU-related behavior changes vary widely [28]. Understanding the potential public health consequences of PIU among young adults pursuing higher education and conducting PIU screenings on university students are therefore clinically significant [29]. Thus, the aim of this study was to identify the sociodemographic, academic, behavioral, and health-related variables associated with PIU among University of Gondar undergraduate students.

## Methods and materials

### Data sources, study setting, design, and period

This study was conducted based on data that had been collected for another purpose. This cross-sectional study was employed from June 1–20, 2022, at the University of Gondar. All five campuses (Science Amba, Atse Tewodros, Fasiledus, Maraki, and Tseda) of the University of Gondar were involved. A total of six colleges, two institutes, two faculties, and one school are found in all campuses, offering 56 undergraduate and 64 postgraduate programs [30].

### Population

All regular undergraduate students at the University of Gondar were the source population. Those students who had registered for the 2022 academic year and available during the data collection period were included in the study population. However, students with thyroid disease and below the age of 18 were excluded.

### Sample size determination

The sample size required for structural equation modeling (SEM) is dependent on the complexity of the model. As a general rule of thumb, the minimum sample size should be no less than 5–20 times the number of parameters to be estimated [31]. Even if the data were collected for another purpose, we checked whether our sample was sufficient to estimate the parameters specified in the hypothesized model. Accordingly, there were 34 observed endogenous variables (6, 9, 9, 7, and 3 for ADHD, depression, problematic internet use (PIU), insomnia, and social support respectively). Therefore, 29 path coefficients were needed since five of them were fixed to 1, in order to give the latent variable measurement scale. One disturbance term for PIU and 34 error terms for those observed endogenous variables. There were 30 exogenous variables (26 observed and four latent) in the specified model. Therefore, the total number of free parameters to be estimated is equal to

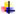
 34*2–5 = 63 path coefficients and error terms for latent variables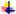 30*2 = 60 path coefficients and variance for exogenous variables and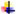 **1** disturbance term for PIU, making a total of 124 free parameters to be estimated. Therefore, the N:q ratio becomes approximately 12:1. Thus, we can conclude that the sample size is sufficient to estimate the parameters specified in the hypothesized model

### Sampling procedure

A stratified simple random sampling technique was applied to select the study participants. A detailed description was written and published elsewhere [32].

### Variables of the study

#### Outcome variables

Problematic internet use (latent endogenous variable).

#### Independent variables

**Latent exogenous variables** (insomnia, ADHD, depression and social support)

#### Observed exogenous variables such as;

**Socio demographic factors:** sex, age, marital status, monthly allowance, maternal education, father education, prior residence, self-rated family economic level and presence of partner.

**Health related factors:** history of chronic disease, history of head injury, history of parental psychiatric illness, birth order and history of stressful life events.

**Behavioral factors:** history of alcohol use, current alcohol use, cigarette smoking, chat chewing, cannabis use, and physical exercise

**Academic related factors:** year of study, number of studying hours per day, number of sleeping hours per day, history of academic failure, worry about academic performance and department type.

### Measurement and data collection tool

In addition to questions about PIU, ADHD, insomnia, social support and depression, questions about behavioral, clinical, academic, and sociodemographic aspects were also included.

#### Problematic internet use

A simplified version of the long PIU-18 item, the Problematic Internet Use Questionnaire-9 (PIUQ-9), was used to measure it. Nine questions total, with five alternative answers: 1 for "never," 2 for "rarely," 3 for "sometimes," 4 for "often," and 5 for "always/almost always" that can be found elsewhere [33, 34]. Higher scores indicate a serious internet use issue.

#### ADHD symptom

The six-item Adult Self-Report Scale-V1.1 (ASRS-V1.1), a simplified screener from the World Health Organization Composite International Diagnostic Interview, was used to measure it. There are five options for each question: never, rarely, sometimes, often, and very often. An increased risk of ADHD symptoms is indicated by higher scores [35].

#### Depressive symptom

It was determined by the Patient Health Questionnaire-9 (PHQ-9) tool. It has nine questions that ask about the frequency of the occurrence of symptoms. All can be scored from zero to three as not at all, several days, more than half the days, and nearly every day, respectively. There can be a minimum score of 0 and a maximum of 27. Higher scores indicate a high risk of depressive symptoms [36].

#### Insomnia

The measurement was made using the insomnia severity index, which has seven questions that ask about the last two weeks of insomnia problems. It has 5 alternatives, which can be scored from 0 to 4, and higher sores indicate a higher risk of insomnia [37].

#### Social support

It was evaluated with the Oslo Social Support Scale (OSSS-3), which consists of items. Four options, numbered 1–4, are provided for the first question; five options, numbered 1–5, are provided for the remaining questions. Larger scores on the OSSS-3 suggest stronger social support [38].

**Ever-smoking** was assessed by asking a single question: “Have you ever smoked cigarettes in your lifetime?” and the responses were “yes” or “no” [39].

#### Physical exercise

Exercising or engaging in any sport, such as walking for at least 20 minutes per day, to which the answers were either "yes" or "no" [40].

**History of alcohol use** was assessed by asking a single question: “Have you ever drunk at least one of the alcoholic beverages (beer, wine, whiskey, Areki, Tela, Tej, etc.) for nonmedical purposes?” and possible responses were “yes” or “no” [41].

**Current alcohol use** was assessed by asking a single question: “Have you ever drunk at least one of the alcoholic beverages (beer, wine, whiskey, Areki, Tela, Tej, etc.) for nonmedical purposes in the last three months?” and possible responses were “yes” or “no” [41, 42].

**Chat chewing** was assessed by asking a single question: “Have you ever chewed chat during your lifetime?” and possible responses were “yes” or “no” [41].

**Cannabis use** was assessed by asking a single question “Have you ever used cannabis during your lifetime?” and possible responses were “yes” or “no” [43].

**The history of parental psychiatric illness** was assessed using one question: “Do your parents have a history of any medically confirmed psychiatric disorders? (It can be any type). Possible responses were “no”, “father”, “mother”, and “both” [32].

### Data collection procedure

A standardized, self-administered questionnaire was used to collect data. The data was collected by four trained data collectors. Following the selection of an identification number for a student using Excel, data collectors located the relevant participants. The questionnaire was given out after the purpose, advantages, and disadvantages of the study were discussed and students gave their consent to participate. The data collector and the principal investigators promptly verified the completeness and consistency of the responses. The principal investigators had direct oversight of every process.

### Data quality assurance and management

Before actual data collection, a pilot study was carried out, and senior and practicing psychiatrists verified the instrument’s face validity. A widely spoken Amharic language, which is also Ethiopia’s official working language, was used to translate the original English questionnaire, and then another person translated it again to English to ensure proper translation of terminologies. Two days of training were given to data collectors regarding the subject matter, confidentiality, ethical behavior, and data collection methods. The researchers kept in regular, timely communication with the collectors throughout the data collection process to address any doubts or concerns regarding the procedures and offer assistance as needed. Early corrective action was taken once the collected data were promptly examined for consistency and completeness. Consistency, any entry errors, missing values, and outliers were carefully reviewed during the data entry process by referring back to the questionnaire.

#### Reliability and validity of tools

To validate the tool, 156 students from Debre Markos University participated in an external pilot study. To further verify the factor loadings, internal consistency, construct, and statistical validity, confirmatory factor analysis was performed. Composite reliability (CR) was used to verify internal consistency. As a result, all constructs’ composite reliability falls between 0.64 and 0.9. As a result, every construct satisfied the required threshold of 0.60 [44]. Average variance extracted (AVE) and CR were used to verify construct validity, also known as convergent validity. Consequently, the AVE falls between 0.38 and 0.49. Despite AVE being less than 0.5, the tool’s convergent validity held true. We can determine that the construct’s convergent validity is sufficient based only on CR, as AVE represents a more conservative estimation of the measurement model’s validity [44, 45]. A thorough explanation was composed and released in another location. A detailed description was written and published elsewhere [32].

### Data processing and analysis

Epi-Data Version 4.61 was used to enter the data. For analysis, Amos SPSS version 21 and STATA version 16 were utilized. We verified a number of assumptions, including multivariate normality, sphericity, sample adequacy, missing data, and outliers. A descriptive analysis and then SEM were employed to test and estimate complex relationships between variables. The adjusted regression coefficients with a 95% confidence interval (CI) and a corresponding p-value < 0.05 were used to interpret the degree of association. Model specification, identification, parameter estimation, model evaluation, and modification were the five logical steps in the model-building process [46].

#### 1. Model specification

**Fig 1:**
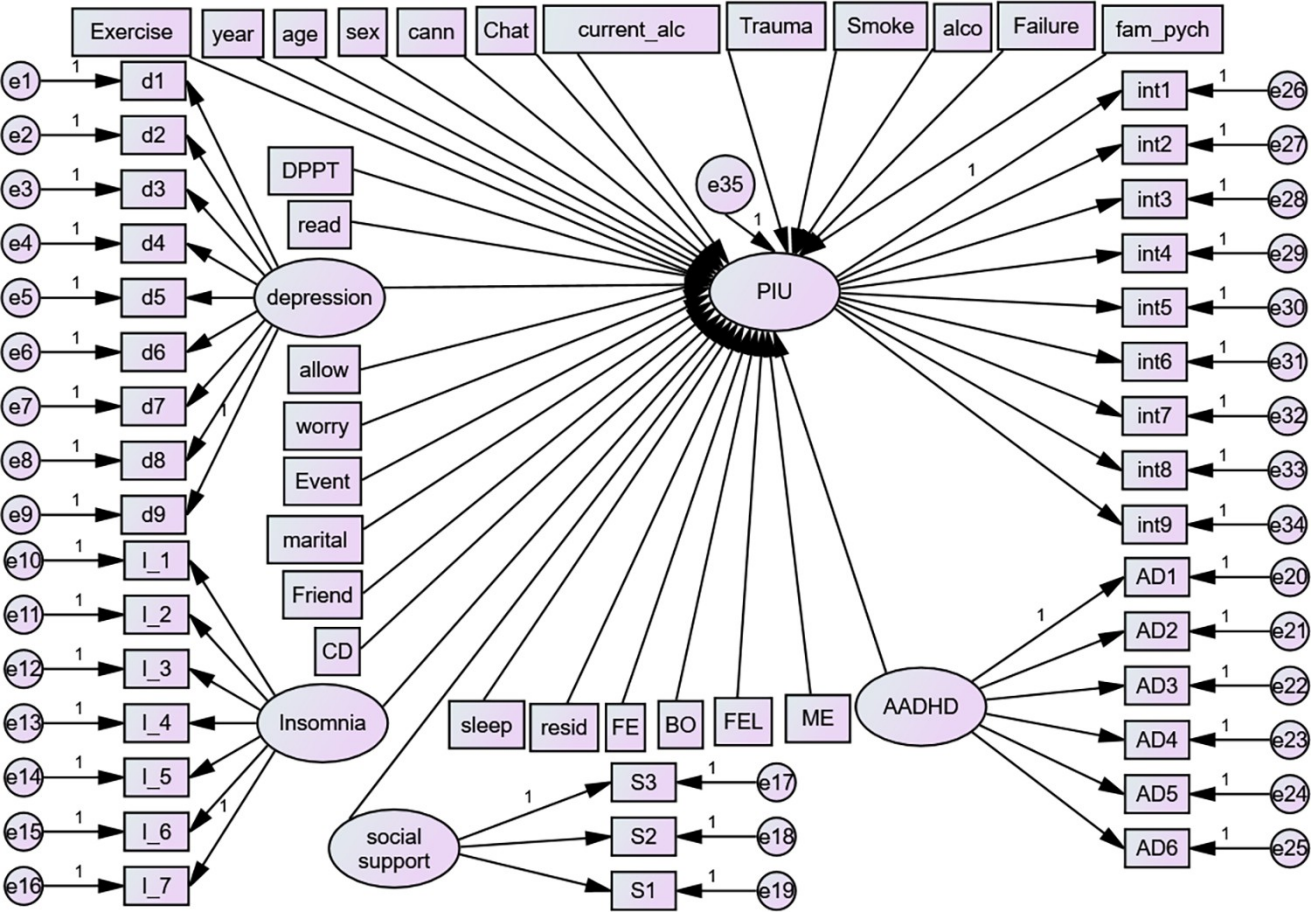
Hypothesized structural model, UoG, Northwest Ethiopia, 2022. Circles indicate latent variables or error terms or disturbances, rectangles indicate observed variables, single arrows indicate factor loadings or regression coefficients, and double arrows indicate the covariance between latent variables; ADHD = Adult Attention Deficit Hyperactivity Disorder, d1-d9 –depression items, AD1-AD6 = ADHD items, S1-S3 = items for Social Support, I_1 –I_7- Insomnia Items, exercise = Physical exercise, year = year of study, cann = cannabis, current_alco = current alcohol, alco = alcohol use, failure = history of academic failure, fam_pych = history of family psychiatric illness, allow- Monthly Allowance, worry = worry about academic performance. Event = history of stressful life event, FEL = family economic level, read and sleep = reading and sleeping hours per day, FE = father education level, ME = mother education level, BO = birth order, PIU = Problematic Internet Use, int1-9 = items for Problematic Internet Use, DPTT = department type and CD = Chronic Disease.

In order to ascertain each relationship and parameter in the model that is relevant to the researcher, the model specification is the initial phase in which the researcher identifies the concept and describes the proposed relationships among the variables [47]. In our case, we hypothesized that all the independent variables mentioned in the variable section were related to PIU (**Fig 1**).

#### 2. Model identification

In order to obtain a solution, the number of free parameters to be estimated, Q, must be equal to or less than the number of non-redundant elements in the sample covariance matrix (Q ≤ P*), where P* = K(K + 1)/2 and K is the number of measured variables. There are three types of model identification. Under-identified: If the degree of freedom (df) is less than zero, just-identified: if the degree of freedom (df) is zero, and over-identified: the degree of freedom will be positive (> 0), and all Q parameters can be estimated, df = (P*—Q) [48]. Model coefficients can only be estimated in the just-identified or over-identified model [47]. In our study, there were 50 observed endogenous and exogenous variables: (50(50+1))/2 = 2550/2 = 1275, non-redundant elements in the sample covariance, and Q = 124. Thus, df = 1275–124 = 1151, which indicates that the model is over-identified.

#### 3. Model estimation

It is a process of finding numerical values for unknown (free) parameters [49]. In this study, the data didn’t satisfy the multivariate normality assumption, so the maximum likelihood estimation approach with 3500 bootstrap samples was used.

#### 4. Evaluation of model fit (model testing)

The fit indices in SEM are used to evaluate each path coefficient. A few indices that are less affected by sample size are the root mean square error of approximation (RMSEA) and comparative fit index (CFI) [47, 50, 51], and the Tucker-Lewis index (TLI) [52]. Models with the least amount of overfitting can be chosen using information criteria (AIC and BIC); however, they are not helpful for testing null hypotheses, even if BIC is another measure of a model’s parsimony among potential models [53]. Therefore, RMSEA, CFI, TLI, AIC, and BIC were used. For each fit index, there are recommended values that should be followed, although none are absolutes [53]. CFI and TLI (NNFI) > 0.90 [47, 50, 51], and RMSEA < 0.05 [51] suggest improved model fitness.

#### 5. Model modification

It entails either fixing or freeing parameters that were free in order to modify a stated and estimated model. To help choose which parameters to add to a model to improve the model fit, the modification index (MI) and the standardized expected parameter change (SEPC) were used. If fixed parameters were linked to a high MI value (> 4) with one degree of freedom and a 0.05 alpha level, they could be included in the model and freely estimated.

### Ethical consideration

The UoG institutional review board granted ethical clearance under reference number Ref No. /IPH/2131/2014. A list of student ID numbers was gathered with permission from the main registrar’s office of the University of Gondar. To collect student data at any moment, a permission letter was requested from every college, institute, faculty, school, or department. Before the questionnaires were distributed, each participant provided written informed consent. During the data gathering process, no personal identifiers were recorded, and the remaining acquired information was stored in a manner that guaranteed data confidentiality and anonymity. The participants were made aware of their right to withdraw from the study at any moment and that their participation was entirely voluntary.

## Result

### Socio-demographic factors

A total of 1514 students participated in this study. Of the participants, males represented almost a third (65%). The participants’ median age was 21 years old (IQR = 2).The majority of respondents (96.9%) were single in their marital status. Roughly a third (66.8%) were urban residents before. More than three-fourths (79.7%) of respondents said they have a stable, intimate partner. About 30.6% and 36.8% of the participant’s mother and father were unable to read and write and attended higher education, respectively. Regarding health and behavioral-related factors, a month before the data was collected, over half (58.5%) of the study participants said they had gone through stressful situations. According to the majority (91.7% and 92.3%) of participants, none of their parents had ever experienced mental health issues or chronic diseases, respectively. The majority of participants (93.4% and 89.8%) indicated that they had never chewed chat or smoked cigarettes, respectively. The median birth order was 2 (IQR = 3). The majority (90.4%) of the participants had not experienced a previous head injury. About two-thirds (66.5%) and half (50.8%) of the participants revealed that they had a history of previous alcohol use and were currently using alcohol, respectively. Almost all (96.9%) and about three-fourths (74.4%) had never used cannabis or regular physical exercise, respectively. The mean score of the PIU and social support scores was 23.2 and 10.3 with a standard deviation of 8.4 and 2.2, respectively. The median scores for ADHD symptoms, depressive symptoms, and insomnia were 12, 8, and 7 with an IQR of 7, 8, and 7, respectively. Regarding factors related with education, more than half (58.7%) of respondents said they were concerned about their academic performance. One-fourth of the responders were from the College of Medicine and Health Science, while the College of Social Science and Humanities accounted for roughly one-fifth. The median hours of study and sleep were likewise recorded at 5 and 8, respectively. For more information, you can refer to the previous work [32].

### Assumptions for structural equation modeling

#### Omitted variable bias

When a certain regression model leaves out a third variable that has the potential to influence both the independent and dependent variables in the causal pathway, it can lead to a condition known as "omitted variable bias" (OVB) [54]. As a result, we made sure that no independent PIU-affecting variable was overlooked that wasn’t included in the model. In order to make sure that the omitted variables aren’t leading to model misspecification, we performed a regression specification-error test (RESET). The results showed that there were no issues with the omitted variables in our study because the P-value was 0.39 > 0.05, indicating that the model has no OVB.

#### Sample size adequacy and sphericity

We evaluated the results of Bartlett’s test for sphericity and the Kaiser-Meyer-Olkin (KMO) measure of sample adequacy. Excellent partial correlation, as indicated by the overall KMO (0.90), and a significant Bartlett’s test of sphericity. A detailed description was written and published elsewhere [32].

#### Multivariate normality, missing and outliers

It was evaluated using the Henze-Zirkler, Doornik-Hansen, and Mardia’s skewness and kurtosis tests, and the results were unsatisfactory. Using the Mahalanobis distance (probability < 0.001 [55]), outliers were examined, and 77 observations show that there is an outlier. Errors in data entry and measurement were verified. All the measurements, though, made sense, and we thought they were all good outliers. Ten observations had values that were missing. Because they made up less than 5% of the sample as a whole and were deemed to be absent entirely at random, a list-wise deletion was carried out.

#### Independence of observation

When clustering at the departmental and college levels was examined, the ICC was significantly below the necessary threshold (0.1) **(S1 Table)**.

#### Common method bias (CMB)

An ordinal scale was used in our study to measure the outcome variables (PIU) and additional exogenous latent factors (social support, insomnia, ADHD, and depression). The Harman single-factor test for CMB was performed. The results showed that the variance explained by a single factor was 19%. Therefore, we can conclude that there was no CMB problem in our study.

#### Confirmatory factor analysis (CFA)

**Fig 2:**
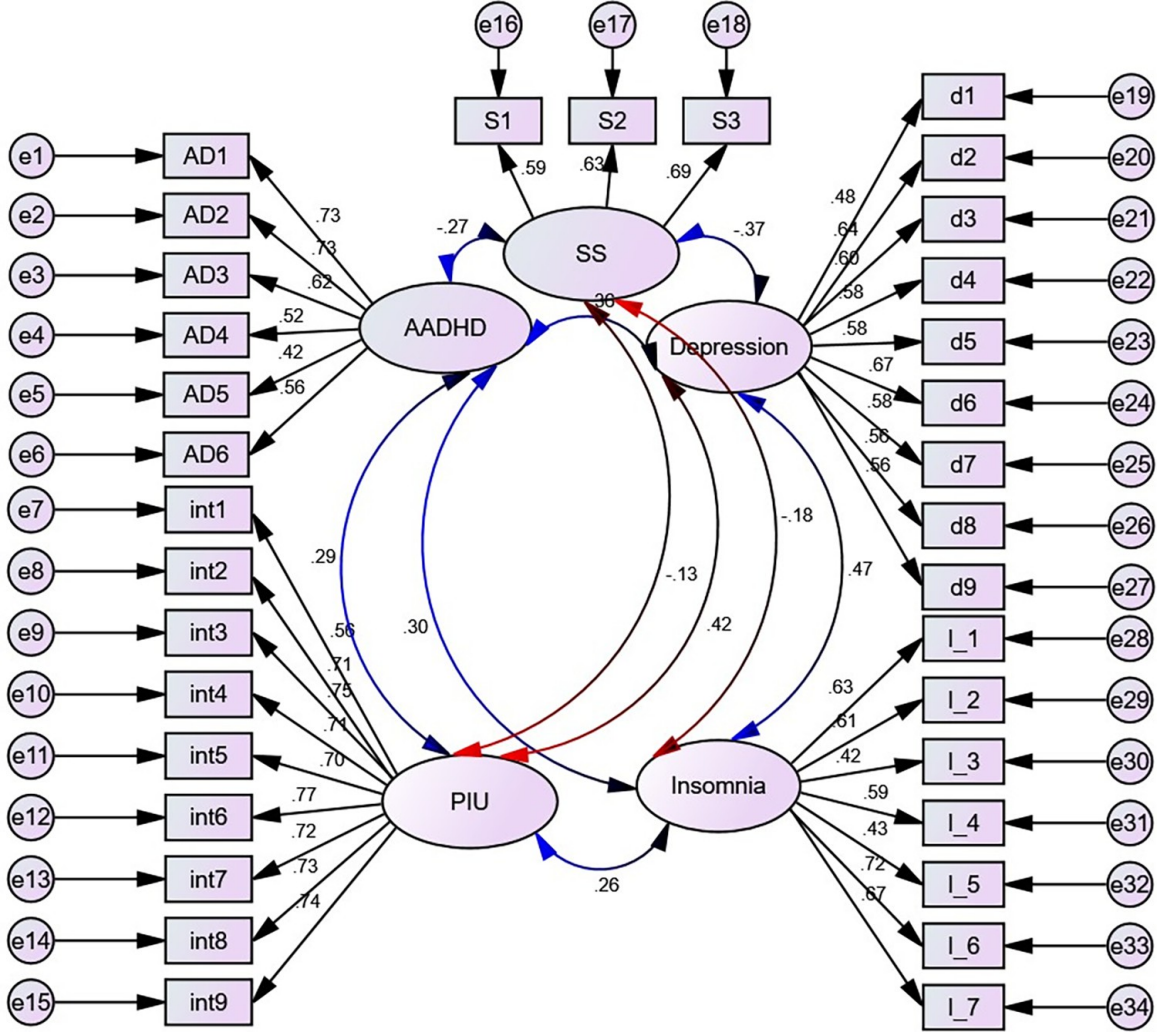
Initial measurement model with standardized estimates displayed, UoG, Northwest Ethiopia, 2022. Circles indicate latent variables or error terms, rectangles indicate observed variables, single arrows indicate factor loadings or regression coefficients, and double arrows indicate the covariance between latent variables.

First, CFA was used to assess measurement models for proposed and empirically confirmed factor structures in our SEM (**Fig 2**). If it is thought that items have common sources other than the latent factors, residuals and errors have been permitted to correlate. We then merged the structural path models and CFA models to create our generic SEM framework.

The measurement model’s model fit indices (TLI = 0.87, CFI = 0.88) fell below the needed cutoff point of 0.9, despite other goodness of fit metrics (CMIN/DF = 4.99, RMSEA (P-value) = 0.05 (0.10)) meeting the necessary threshold. Additionally, the BIC was 2741.96 and the AIC was 2738.24. This suggests that the sample variance-covariance data does not support the analysis and that the CFA of the original models is inadequate. As a result, we kept adding potential adjustments depending on MI and SEPC. In construct indicators, covariance between error terms was included (**S2 Table and S1 Fig**). All of the model fit metrics (CMIN/DF = 2.97, TLI = 0.93, CFI = 0.94, RMSEA (P-value) = 0.04 (1.00), AIC = 1678.48, and BIC = 1682.73) thus become acceptable. But the model wasn’t parsimonious. Consequently, a type of parceling called average partial factorial parceling was used.

*Item parceling*. Three constructs, ADHD, insomnia, and depression, were subjected to average partial factorial parceling. The standard recommendation is three parcels [56]. Hence, every construct had three parcels (**S3 Table**). In terms of model fitness, CMIN/DF = 7.24, TLI = 0.91, CFI = 0.92, RMSEA (P-value) = 0.06 (0.00), AIC = 1400.3, and BIC = 1676.75 were the majority of model fit indices at the necessary level. But in order to raise the model fit indices, we added more potential adjustments based on MI and SEPC (**S2 Fig**). In conclusion, every goodness of fit index (CMIN/DF = 3.47, TLI = 0.96, CFI = 0.97, RMSEA (P-value) = 0.04 (1.00), AIC = 711) fell within an acceptable range. BIC, a measure of model parsimony, dropped dramatically from 1676.8 to 713.8. As a result, we chose to move on with the adjusted measurement model that included parceled indicators.

### Structural model

**Fig 3:**
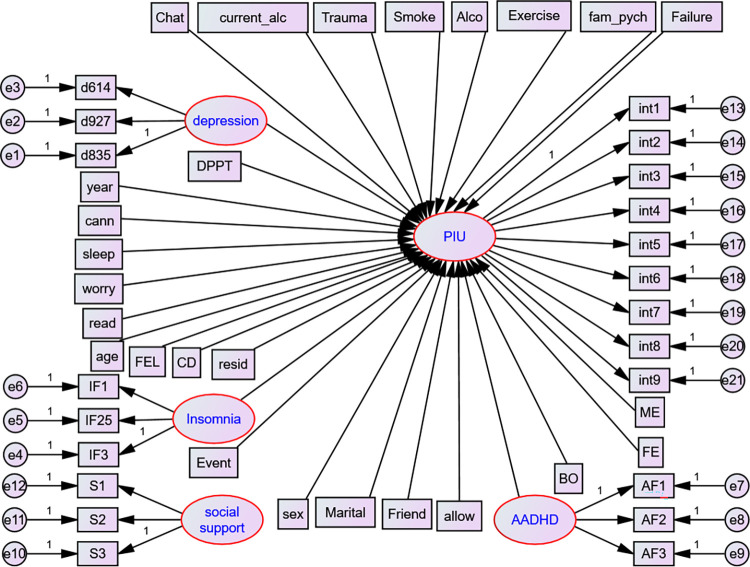
Full structural model after parceling, UoG, Northwest Ethiopia, 2022. *AF1-3- represents parcel 1–3 for ADHD*, *IF1*, *1F25*, *IF34- represents parcel 1–3 for insomnia and d614*, *d927 and d835 represents parcel 1–3 for depression respectively*; *exercise = Physical exercise*, *year = year of study*, *cann = cannabis*, *current_alco = current alcohol*, *alco = alcohol use*, *failure = history of academic failure*, *worry = worry about academic performance*. *Event = history of stressful life event*, *read and sleep = reading and sleeping hours per day*, *ME = mother education level*, *BO = birth order*, *PIU = Problematic Internet Use*, *int1-9 = items for Problematic Internet Use*, *DPTT = department type and CD = Chronic Disease*.

An analysis of a set of relations between variables can be done using a statistical technique called a structural model. Variables can be both dependent and independent variables (**Fig 3)**.

#### Model selection

Prior to allowing correlation between independent predictors, insignificant variables were eliminated from the model. After that, error terms for the indicators within the same construct were added. Fit indices and information criteria were used to compare models **(Table 1)**.

**Table 1:** Model selection after parceling, UoG, Northwest Ethiopia, 2022.

Model	AIC	BIC	CFI	TLI	RMSEA (P-value)
Hypothesized model	11675.3	12185.7	0.49	0.47	0.08(0.00)
Model with significant factors	1894.5	2133.8	0.88	0.87	0.08(0.00)
Model with significant factors and error terms covariance.	1136.9	1429.3	0.94	0.93	0.06(0.00)
Model with significant factors and covariance between error terms and exogenous variables.	815.4	1123.7	0.96	0.95	0.045(0.99) **Selected**

### Factors associated with PIU

**Fig 4:**
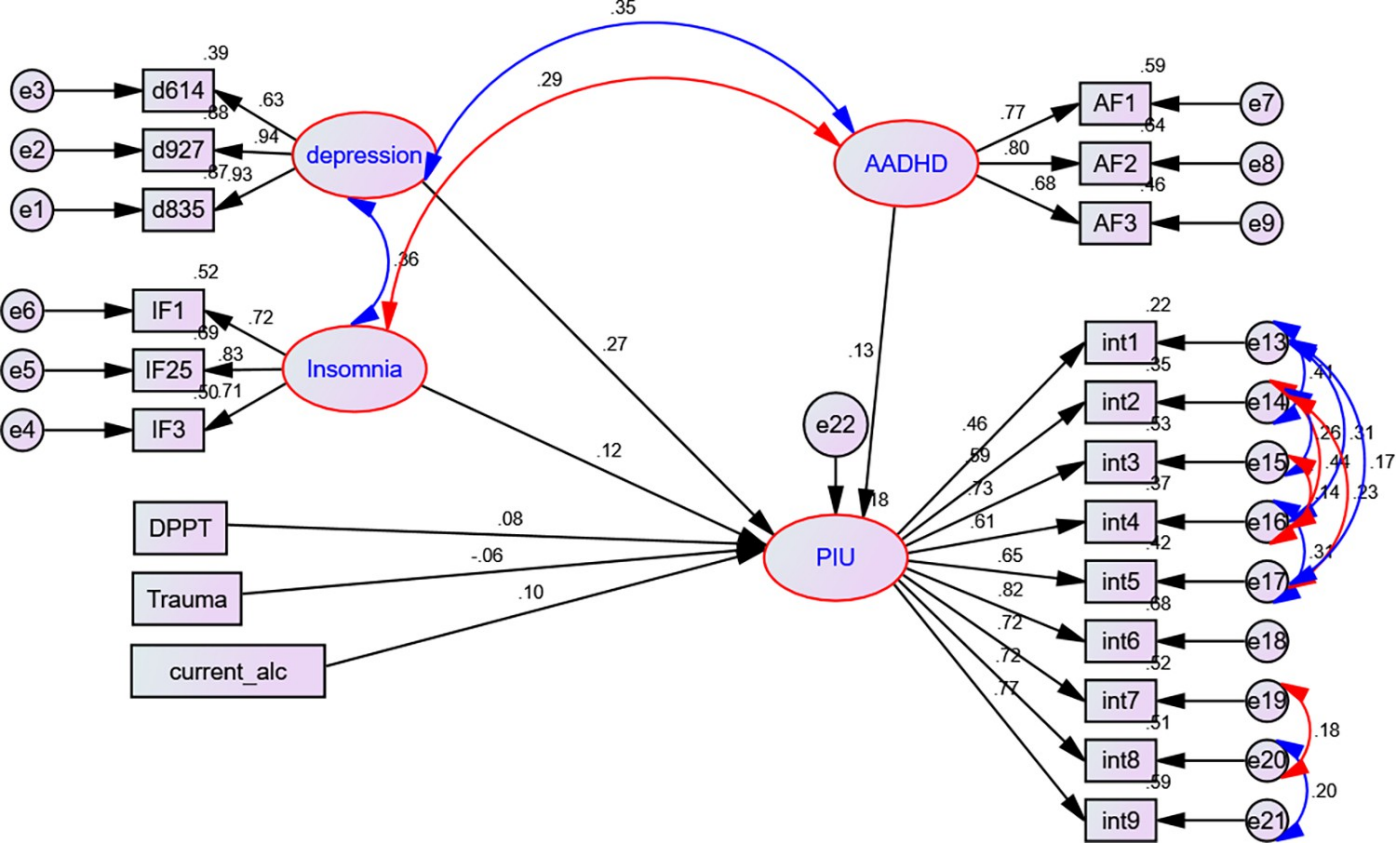
Final SEM showing factors associated with PIU, UoG, Northwest Ethiopia, 2022. Standardized estimates are displayed.

The model was modified to exclude variables such as sex, age, residence, average monthly allowance, marital status, year of study, cannabis use, smoking history, family history of mental health issues, physical activity history, academic failure history, and self-reported household income level, worry about academic performance, presence of a stable partner, history of stressful events, presence of medically confirmed chronic disease, history of smoking and chat chewing, sleeping and reading time per day, social support, previous history of alcohol use, father and mother education, and birth order by considering their statistically insignificant effect on PIU. Once the path coefficients have been adjusted and all the remaining variables have been controlled, the model is summarized (**Fig 4)**.

Our final model indicated that department type, current alcohol use, history of head injury, depressive symptoms, insomnia symptoms, and ADHD were significant factors. Thus, being from non-health departments [adjusted β = 0.11, 95% CI: 0.037, 0.181], current alcohol use [adjusted β = 0.12, 95% CI: 0.061, 0.187], depressive symptoms [adjusted β = 0.23, 95% CI: 0.175, 0.291], insomnia symptoms [adjusted β = 0.12, 95% CI: 0.060, 0.196], and ADHD symptoms [adjusted β = 0.11, 95% CI: 0.049, 0.166] had a significant positive effect on PIU. Having a history of head injury had a significant negative effect [adjusted β = -0.12, 95% CI: -0.226, -0.021] on PIU (**Table 2**).

**Table 2:** Factors associated with PIU among University of Gondar undergraduate students, northwest Ethiopia, 2022. Unstandardized estimates.

Variable	Adjusted estimates [95% CI]	Standard errors	P—Value
Current alcohol use	0.12 [0.061, 0.187]	0.033	0.001
Department type	0.11 [0.037, 0.181]	0.036	0.003
History of head injury	-0.12 [-0.226, -0.021]	0.053	0.021
Depressive symptoms	0.23 [0.175, 0.291]	0.029	0.000
Insomnia symptoms	0.12 [0.060, 0.196]	0.034	0.000
ADHD symptoms	0.11 [0.049, 0.166]	0.030	0.001

^**a**^ CI—Confidence Interval

## Discussion

This study applied SEM to investigate factors associated with PIU among University of Gondar undergraduate students in Northwest Ethiopia. And our final reduced structural equation model revealed that depressive symptoms had a positive effect [β = 0.23, 95% CI: 0.175, 0.291] on PIU. Which indicates students with high depressive symptom scores had a high PIU score as compared to their counterparts, keeping other variables in the model controlled. This finding was supported by previous reports [5, 13, 17, 23, 25, 27, 57–62]. This might be due to the fact that students with depressive symptoms may use the internet and other social media to relieve their loneliness, emotional distress, and unhealthy lifestyle [9, 63].

Moreover, insomnia symptoms were also positively associated with a higher PIU score [β = 0.12, 95% CI: 0.060, 0.196] in our study. Which indicates students with high insomnia symptom scores had a high PIU score as compared to their counterparts. In addition, a report from China and Palestine [35, 64] also revealed a strong positive association between PIU and insomnia. This could be the result of spending more time online, as using a computer at night can raise arousal levels and disrupt the restorative processes needed to fall asleep [65–68]. This interaction and relationship between these disorders may indicate a bidirectional relationship between them [69].

Being a non-health department student had a positive association [β = 0.11, 95% CI: 0.037, 0.181] with PIU. This indicates that being from a non-health department increases the level of PIU by 0.11 compared to their counterparts. This might be due to the fact that students from non-health departments have more free time to spend on social media, which can increase the likelihood of developing PIU [9]. Clinical rotations, classes, and exam preparation are all common parts of medical students’ hectic schedules. Particularly on weekends and during the pauses between semesters, they have some spare time [70]. Another study conducted in Argentina also revealed that the majority of medical students’ free time was spent on social and cultural activities, sports activities, sharing time with friends, and being with family [71].

Moreover, symptoms of ADHD have a significant positive effect on PIU [β = 0.11, 95% CI: 0.049, 0.166]. This indicates students with higher ADHD symptoms scores had a higher PIU level compared to their counterparts, keeping other variables in the model controlled. This finding is supported by a systematic review [72–74], a study conducted in Britain [75], New York [61], Japan [76], Turkey [77, 78], and Malaysia [79]. This can be due to the fact that individuals with ADHD are more sensitive to rewards. People with ADHD can greatly benefit from the motivating payoff that comes from feeling in control and having the opportunity to express themselves online [80]. In addition, compulsive and risky habits, such as problematic internet use, have a high tendency to cause comparable dopamine release and euphoric sensations in people with ADHD [81].

Current alcohol use was significantly associated with the PIU score [β = 0.12, 95% CI: 0.061, 0.187]. This indicates current alcohol user students had an increased PIU score compared to their counterparts. Previous findings also indicated a close relationship between alcohol use and PIU [4, 82–85]. Furthermore, our research demonstrated that insomnia significantly increased PIU. This could be because alcohol drinkers may have a stronger dependence on the internet, and insomnia may be a symptom of internet addiction. There is also a complex link between alcohol consumption, insomnia, and PIU. Extensive research on adolescents in South Korea and Japan has also shown that adolescents who drink alcohol have a greater likelihood of sleep disturbance [82, 86, 87].

Our study also revealed a negative relationship between history of head injury and PIU [β = -0.12, 95% CI: -0.226, -0.021], where students with a history of injury had a decreased PIU compared to their counterparts, keeping other variables in the model controlled. This may be as a result of the long-term cognitive issues that can arise from head traumas, such as issues with memory, concentration, and problem-solving skills, which can then lead to emotional or learning challenges [32, 88, 89]. Thus, students with these problems may be forced to spend their free time on their education to cope with the highest burden in higher education institutions rather than spending their time on the internet. In contrast to this finding, a study conducted in China revealed a positive association between childhood trauma and PIU [90]. This difference might be due to the fact that the previous study included other types of trauma, like emotional and psychological trauma, in addition to physical trauma, which the current study didn’t address.

Our findings imply that public health policy planners may benefit from taking into account university students’ internet usage and how it relates to their mental health state when creating context-based treatments. Additionally, it encourages university administration to step in when inappropriate internet use unfolds.

## Strength and limitation of the study

Our findings indicated the relationship between different mental health problems and PIU and highlighted the mechanisms of the problem in order to understand and inform public health policymakers and planners on internet use. However, this study does not show any cause-and-effect relationship because of the cross-sectional nature of the study design. Moreover, all our reports were based on self-reported data, which may result in socially desirable bias.

## Conclusion

Our final SEM model revealed that factors such as current alcohol use, non-health department type, depressive symptoms, insomnia symptoms, ADHD symptoms, and history of head injury were significantly associated with PIU. Therefore, it is better to support future studies with clinical diagnoses to investigate the relationship between PIU and other behavioral and mental health disorders. Moreover, strategies aimed at the early identification of PIU may lead to an improvement in the psychosocial health of university students.

## Supporting information

S1 Table ICC result for outcome variables (PIU), UoG, Northwest Ethiopia, 2022. (DOCX)

S2 Table Modification indices among the error terms of the constructs, as covariance between each pair of items during CFA, UoG, Northwest Ethiopia, 2022. (DOCX)

S3 Table Parceling applied for three construct variables, UoG, Northwest Ethiopia, 2022. (DOCX)

S4 Table Participant’s responses on each items of PIU, UoG, Northwest Ethiopia, 2022. (DOCX)

S1 Fig Modified measurement model with standardized estimates displayed, UoG, Northwest Ethiopia, 2022. (TIFF)

S2 Fig Modified measurement model after parceling with standardized estimates displayed, UoG, Northwest Ethiopia, 2022. (TIFF)

## Abbreviations and acronyms

PIU: Problematic Internet Use
AVE: Average Variance Extracted
ADHD: Attention Deficit Hyperactivity Disorder
CFA: Confirmatory Factor Analysis
SEM: Structural Equation Modeling
