# Supplementary material for: Factors associated with problematic internet use among University of Gondar undergraduate students, Northwest Ethiopia: Structural equation modeling
Source: PLoS One. 2024 Jun 18;19(6):e0302033. doi: 10.1371/journal.pone.0302033 (PMC11185474; doi:10.1371/journal.pone.0302033)
Supplement: S1 Table — (DOCX) [file pone.0302033.s001.docx]

**S1 Table: ICC result for outcome variables, UoG, Northwest Ethiopia, 2022.**

| Outcome variables | Clustering variable | ICC [95% CI] |
| --- | --- | --- |
| PIU | College | 0.01 [0.004, 0.04] |
|  | Department | 0.04 [0.02, 0.09] |
