# Supplementary material for: Factors associated with problematic internet use among University of Gondar undergraduate students, Northwest Ethiopia: Structural equation modeling
Source: PLoS One. 2024 Jun 18;19(6):e0302033. doi: 10.1371/journal.pone.0302033 (PMC11185474; doi:10.1371/journal.pone.0302033)
Supplement: S4 Table — (DOCX) [file pone.0302033.s004.docx]

**S4 Table: Participant’s responses on each items of PIU, UoG, Northwest Ethiopia, 2022.**

| PIU | Response | | | | | Total |
| --- | --- | --- | --- | --- | --- | --- |
| Item | Never (1) | Rarely (2) | Sometimes (3) | Often (4) | Always (5) | 1504 |
| PIU 1 | 184(12.2%) | 375(24.9%) | 247(16.4%) | 397(26.4%) | 301(20%) | 1504 |
| PIU 2 | 209(13.9%) | 424(28.2%) | 336(22.3%) | 372(24.7%) | 163(10.8%) | 1504 |
| PIU 3 | 456(30.3%) | 416(27.7%) | 301(20%) | 220(14.6%) | 111(7.4%) | 1504 |
| PIU 4 | 285(18.9%) | 431(28.7%) | 264(17.6%) | 358(23.8%) | 166(11%) | 1504 |
| PIU 5 | 252(16.7%) | 464(30.9%) | 283(18.8%) | 384(25.5%) | 121(8.1%) | 1504 |
| PIU 6 | 519(34.5%) | 412(27.4%) | 301(20%) | 190(12.6%) | 82(5.5%) | 1504 |
| PIU7 | 502(33.4%) | 420(27.9%) | 272 (18.1%) | 209(13.9%) | 101(6.7%) |  |
| PIU8 | 504(33.5%) | 417(27.7%) | 264(17.6%) | 228(15.2%) | 91(6%) |  |
| PIU9 | 531(35.3%) | 440(29.3%) | 245(16.3%) | 190(12.6%) | 98(6.5%) |  |
